# Fatal Outbreak of *Mycoplasma capricolum* Pneumonia in Endangered Markhors

**DOI:** 10.3201/eid1712.110187

**Published:** 2011-12

**Authors:** Stéphane Ostrowski, Francois Thiaucourt, Mulojon Amirbekov, Abdurahmon Mahmadshoev, Lucía Manso-Silván, Virginie Dupuy, Dustmurod Vahobov, Orom Ziyoev, Stefan Michel

**Affiliations:** Wildlife Conservation Society, New York, New York, USA (S. Ostrowski);; Centre de Cooperation International en Recherche Agronomique pour le Développement, Montpellier, France (F. Thiaucourt, L. Manso-Silván, V. Dupuy);; Ministry of Agriculture, Dushanbe, Tajikistan (M. Amirbekov, A. Mahmadshoev, O. Ziyoev);; Academy of Agricultural Sciences, Dushanbe (D. Vahobov);; Nature Protection Team, Dushanbe and Khorog, Tajikistan (S. Michel)

**Keywords:** outbreak, Mycoplasma, goats, animals, wild, conservation of natural resources, tuberculosis and other mycobacteria, Tajikistan, endangered, pneumonia, endangered, markhors

## Abstract

A pneumonia outbreak reduced the numbers of a wild population of endangered markhors (*Capra falconeri*) in Tajikistan in 2010. The infection was diagnosed by histologic examination and bacteriologic testing. *Mycoplasma capricolum* subsp. *capricolum* was the sole infectious agent detected. Cross-species transmission from domestic goats may have occurred.

*Mycoplasma capricolum* subsp. *capricolum* and *M. capricolum* subsp. *capripneumoniae* are closely related subspecies of the *M. mycoides* cluster (*1*). Whereas *M. capricolum* subsp. *capripneumoniae* is the etiologic agent of contagious caprine pleuropneumonia (CCPP), a severe and typically lethal respiratory disease, *M. capricolum* subsp. *capricolum* infection is usually not fatal and instead results in chronic inflammation in a variety of organs, including joints, udder, eyes, and lungs (*2*). *M. capricolum* subsp. *capricolum* infection occurs worldwide and appears widespread but has rarely been found in species of small ruminants other than domestic goats and, more occasionally, sheep (*2**,**3*). This lack of evidence may be partially because few studies have applied sensitive molecular techniques for its detection in nondomestic ruminants (*2**,**3*). Domestic goats can carry *M. capricolum* subsp. *capricolum* asymptomatically, notably in the ear canal (*4*), and pose an insidious risk for cross-species transmission with sympatric wild caprines (*2**,**3*).

## The Study

The markhor (*Capra falconeri*) is an endangered wild goat in a continuous decline; the global population is <2,500 mature animals (*5*). In Tajikistan, <350 animals may survive in fragmented subpopulations in the remote Hazratishoh and Darvaz mountain ranges along the Afghanistan border (*6*). They live sedentarily over relatively small home ranges, moving <5 km per day (*7*). Throughout its range, the markhor has to forage in close proximity to domestic goats (*8*) and is therefore prone to infections of contagious agents transmitted by these animals.

**Figure 1:**
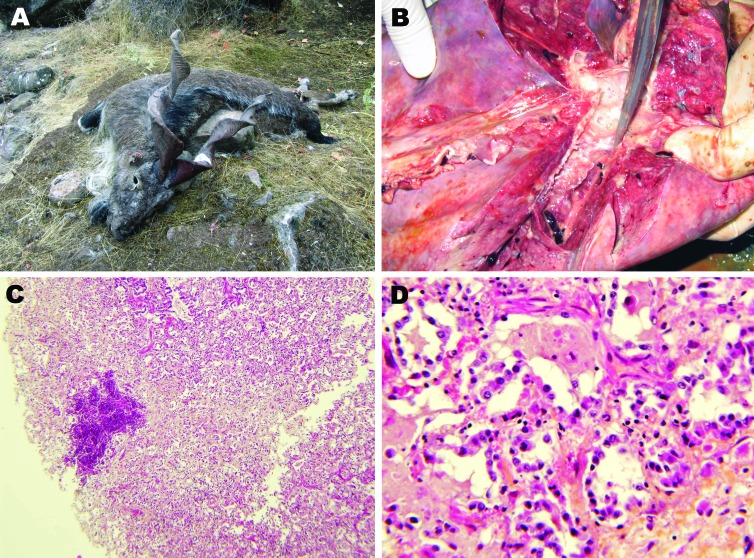
Pneumonia caused by *Mycoplasma capricolum* subsp. *capricolum* in markhors (*Capra falconeri*), Tajikistan, 2010. A) Adult male markhor found dead with signs of pneumonia and no indications of emaciation. B) Disseminated gray areas of consolidation in the cardiac lobe of the right lung with mucopurulent exudate in bronchi. C) Diffuse proliferative interstitial pneumonia associated with a lesion of suppuration (hematoxylin and eosin stain; original magnification ×40). D) Interstitial pneumonia showing fibrotic thickening of alveolar walls and epithelialization of pneumocytes (hematoxylin and eosin stain; original magnification ×250).

During September 17–October 18, 2010, eleven markhors that displayed labored breathing and 64 markhors that had recently died were found in 5 localities (Table) in the district of Shuroabad, Khatlon Province, usually in close proximity to water sources (Figure 1, panel A). All but 4 carcasses were too scavenged for thorough examination in the field, and 1 dying adult female was sent on September 20 to the Republican Veterinary Laboratory in Dushanbe for necropsy. The most relevant necropsy findings noted in the field were the following: an abundant serous to mucopurulent nasal discharge; and, internally, severe pneumonia associated with a variable level of pleural fluid. The female markhor examined in Dushanbe showed gray areas of consolidations in the apical and cardiac lobes of the right lung and yellow pleural fluid (Figure 1, panel B). The cut surface of the affected lobes revealed a fine granular texture, and mucopurulent exudate could be expressed from the bronchi. The joints, eyes, and udder were not affected. Regarding indications of CCPP, although gross lesions were limited to the thoracic cavity, fibrinous pleurisy (*9*) was not observed. The histopathologic findings were consistent with proliferative interstitial pneumonia associated with multifocal suppurations (Figure 1, panel C). Findings included diffuse thickening of the interlobular septa, alveolar epithelialization, and polymorphonuclear leukocytes in alveolar and bronchiolar spaces (Figure 1, panel D). Also, disseminated and abundant neutrophil infiltration of alveolar spaces was found that, when ruptured, created multifocal nodules. The left lung was congested.

**Table:** Geographic distribution of dead markhors (*Capra falconeri*) during outbreak of pneumonia, Tajikistan, 2010

Locality	UTM coordinates*	No. deaths†
Obidara	42N 584745 4163741	23‡
Shulashdara	42N 584415 4161151	7
Siyorish	42N 587145 4163989	13
Pamdara	42N 585515 4159600	8
Dudara	42N 589488 4163693	13
Total		64

*UTM, universal transverse mercator. Source: World Geodetic System 84. †Minimum number. ‡13 adult males, 6 adult females, 4 juveniles (<1 year of age).

**Figure 2:**
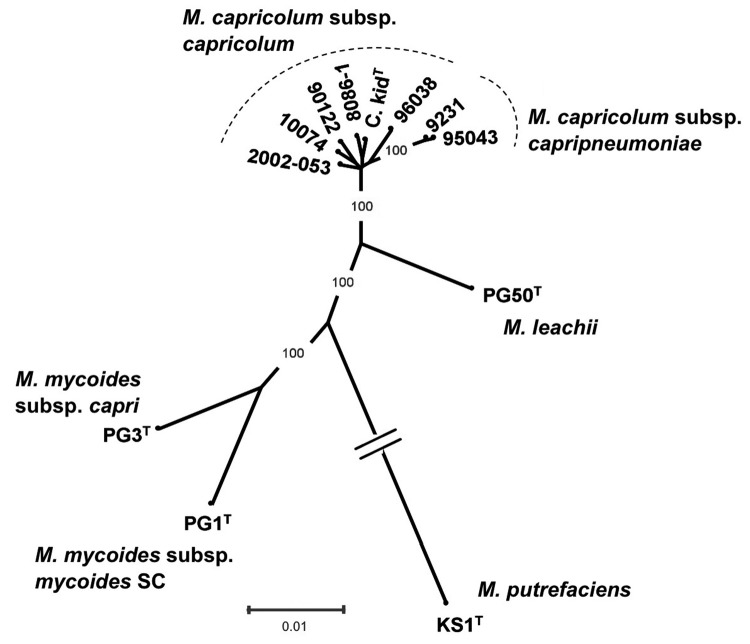
Phylogenetic tree of the *Mycoplasma mycoides* cluster, including the isolate from markhor (*Capra falconeri*) 10074 (1.4) in Tajikistan, 2010, together with available *M. capricolum* subsp. *capricolum* strains, as well as type strains corresponding to other species or subspecies from this cluster and *M. putrefaciens*, used as outgroup. The tree, derived from distance analysis of 5 concatenated protein-coding sequences (*fusA*, *glpQ*, *gyrB*, *lepA*, *rpoB*), was constructed by using the neighbor-joining algorithm. Bootstrap percentage values were calculated from 500 resamplings, and values >90% are indicated. Scale bar indicates distance equivalent to 1 substitution per 100 nt positions. Note that the branch corresponding to the outgroup has been shortened, as indicated by 2 parallel bars.

No bacteria were isolated from pleural fluid that was obtained aseptically and inoculated onto 5% sheep blood agar. However, when a healthy 5-month-old domestic goat (*C. hircus*) was inoculated intratracheally with 5 mL of this pleural fluid, pneumonia developed within 8 days. To evaluate the possibility that the outbreak might have been caused by *M. capricolum* subsp. *capripneumoniae*, which was first identified in Tajikistan in 2009 (*10*), samples were sent to Centre de Coopération International en Recherche Agronomique pour le Developpement, Montpellier, France, a reference laboratory for CCPP for the World Organisation for Animal Health. Pleural fluid from the markhor was centrifuged at low speed (500 *g* for 10 min) to eliminate inflammatory cells, and DNA was extracted from the supernatant with the DNeasy blood and tissue kit (QIAGEN, Courtaboeuf, France), according to the manufacturer’s instructions. Real-time PCR (*11*), specific for *M. capricolum* subsp. *capripneumoniae*, provided negative results, whereas a partial 16S rRNA gene sequence could be amplified and sequenced (*12*). BLAST analysis (http://blast.ncbi.nlm.nih.gov/Blast.cgi) showed that the sequence corresponded to that of a mycoplasma from the *M. mycoides* cluster. Markhor pleural fluid and lung tissue from the domestic goat were then injected into modified Hayflick broth (*1*) and onto agar plates and incubated at 37°C in 5% CO_2_. Typical *Mycoplasma* colonies appeared on solid medium after 1–2 days. A clone from the markhor culture, named 10074 (1.4), was used for PCR amplification and sequencing of *fusA*, *glpQ*, *gyrB*, *lepA*, and *rpoB* partial gene sequences as described (*1*). The *fusA* sequence was identical to a previously submitted sequence (GenBank accession no. EF071735). All other sequences were novel and have been assigned new GenBank accession nos.: HQ882179 (*glpQ*), HQ882180 (*gyrB*), HQ882181 (*lepA*), HQ882178 (*rpoB*). The 5 protein-coding sequences were then concatenated and incorporated in the *M. mycoides* cluster phylogenetic tree (Figure 2). The strain clustered with *M. capricolum* subsp. *capricolum* and was therefore identified as belonging to this subspecies. The isolate from the inoculated domestic goat was undistinguishable from 10074 (1.4) by sequencing of the 5 housekeeping genes.

The pattern of inflammation in the right lung in the necropsied markhor was consistent with *M. capricolum* subsp. *capricolum* respiratory infection in domestic goats (*2*), and the presence of *M. capricolum* subsp. *capricolum* in pleural fluid, the lack of findings indicative of alternative etiologic agents, and the reproduction of the disease in the domestic goat with isolation of *M. capricolum* subsp. *capricolum* from the lungs support a causal relationship between *M. capricolum* subsp. *capricolum* and disease in markhors.

## Conclusions

Although because of difficult field conditions, only 1 markhor could be thoroughly investigated, these findings support the hypothesis that *M. capricolum* subsp. *capricolum*, a newly recorded pathogen in free-ranging wild ruminants, may be responsible for the pneumonia epizootic observed in endangered markhors. This outbreak claimed ≈20% of the population remaining in Tajikistan, and more markhor deaths might have remained undetected.

The source of *Mycoplasma* infection in markhors is unknown, but domestic goats, which have contact with markhors, particularly in the summer, might have been responsible for the emergence of *M. capricolum* subsp. *capricolum* in this wild species. In November 2008, a disease resembling CCPP affected domestic goats and, to a lesser extent, sheep in Shuroabad District (<40 km from the area where the dead markhors were found) with a case-fatality rate of 20%–30% (*13*). However, the origin of this outbreak could not be investigated. Although the clinicopathologic features of the disease in the markhors resembled CCPP, *M. capricolum* subsp. *capricolum* was identified as the most probable causative agent. In fact, several mycoplasmas have been associated with respiratory diseases in ruminants, including *M. capricolum* subsp. *capricolum* infection in young domestic goats (*2**,**14*). Further testing should evaluate the presence of sick animals or asymptomatic carriers of *M. capricolum* subsp. *capricolum* in livestock and wildlife in Shuroabad District.

Environmental and nutritional stressors may exacerbate the susceptibility of ruminants to *Mycoplasma* infections (*2**,**15*). The disease appeared at the end of summer, when markhors are forced by livestock and guard dogs to retreat to suboptimal habitats with poor forage (*8*). It was also the end of the dry season, when markhors may have contact with livestock at the few remaining water sources.

Findings from this study support the conclusion that the markhor is vulnerable to *M. capricolum* subsp. *capricolum*. In the generalized context of increasing encroachment of livestock into wild habitats, markhors and other wild caprines might be at risk for future mycoplasmosis outbreaks. This case emphasizes the need for continuous disease surveillance in domestic animals that have contact with valuable wildlife resources.

## References

[1] Manso-Silván L, Perrier X, Thiaucourt F. Phylogeny of the *Mycoplasma mycoides* cluster based on analysis of five conserved protein-coding sequences and possible implications for the taxonomy of the group. Int J Syst Evol Microbiol. 2007;57:2247–58. 10.1099/ijs.0.64918-0 17911291

[2] Frey J. Mycoplasmas of animals. In: Razin S, Herrmann R, editors. Molecular biology and pathogenicity of mycoplasmas. New York: Kluwer Academic/Plenum Publishers; 2002. p. 73–90.

[3] Nicolas MM, Stalis IH, Clippinger TL, Busch M, Nordhausen R, Maalouf G, Systemic disease in Vaal rhebok (*Pelea capreolus*) caused by mycoplasmas in the mycoides cluster. J Clin Microbiol. 2005;43:1330–40. 10.1128/JCM.43.3.1330-1340.2005 15750104 PMC1081266

[4] Cottew GS, Yeats FR. Mycoplasmas and mites in the ears of clinically normal goats. Aust Vet J. 1982;59:77–81. 10.1111/j.1751-0813.1982.tb02731.x 7159311

[5] Valdez R. Capra falconeri. In: International Union for Conservation of Nature 2011, IUCN red list of threatened species [cited 2011 Jan 22]. http://www.iucnredlist.org/apps/redlist/details/3787/0

[6] Weinberg PI, Fedosenko AK, Arabuli AB, Myslenkov A, Romashin AV, Voloshina I, Commonwealth of independent states. In: Shackleton DM, editor. Wild sheep and goats and their relatives: status survey and conservation action plan for Caprinae. Gland (Switzerland): International Union for Conservation of Nature; 1997. p. 172–93.

[7] Baskin L, Danell K. Ecology of ungulates: a handbook of species of eastern Europe and northern and central Asia. Berlin: Springer-Verlag; 2003.

[8] Woodford MH, Frisina MR, Awan GA. The Torghar conservation project: management of the livestock, Suleiman markhor (*Capra falconeri*) and Afghan urial (*Ovis orientalis*) in the Torghar Hills, Pakistan. Game Wildl Sci. 2004;21:177–87.

[9] Thiaucourt F, Bölske G, Leneguersh B, Smith D, Wesonga H. Diagnosis and control of contagious caprine pleuropneumonia. Rev Sci Tech. 1996;15:1415–29. 9190021 10.20506/rst.15.4.989

[10] Office International des Epizooties. Contagious caprine pleuropneumonia, Tajikistan. 2009 May 15 [cited 2009 Nov 3]. http://web.oie.int/wahis/public.php?page=event_summary&reportid=8610

[11] Lorenzon S, Manso-Silván L, Thiaucourt F. Specific real-time PCR assays for the detection and quantification of *Mycoplasma mycoides* subsp. *mycoides* SC and *Mycoplasma capricolum* subsp. *capripneumoniae.* Mol Cell Probes. 2008;22:324–8. 10.1016/j.mcp.2008.07.003 18678244

[12] Botes A, Peyrot BM, Olivier AJ, Burger WP, Bellstedt DV. Identification of three novel mycoplasma species from ostriches in South Africa. Vet Microbiol. 2005;111:159–69. 10.1016/j.vetmic.2005.10.017 16280203

[13] Food and Agriculture Organization. Contagious caprine pleuropneumonia detected for the first time in Tajikistan. 2010 [cited 2011 Jan 28]. http://www.fao.org/docrep/012/i1648e/i1648e00.pdf

[14] DaMassa AJ, Brooks DL, Adler HE, Watt DE. Caprine mycoplasmosis: acute pulmonary disease in newborn kids given *Mycoplasma capricolum* orally. Aust Vet J. 1983;60:125–6. 10.1111/j.1751-0813.1983.tb05912.x 6870714

[15] DaMassa AJ, Wakenell PS, Brooks DL. Mycoplasmas of goat and sheep. J Vet Diagn Invest. 1992;4:101–13. 10.1177/104063879200400126 1554763

